# Facilitating the interpretation of pedobarography: the relative midfoot index as marker for pathologic gait in ankle osteoarthritic and contralateral feet

**DOI:** 10.1186/s13047-016-0177-y

**Published:** 2016-12-01

**Authors:** Frigg Arno, Frigg Roman, Wiewiorski Martin, Goldoni Jennifer, Horisberger Monika

**Affiliations:** 1 Orthopedic Department, University of Basel, Basel, Switzerland; 2 Department of Philosophy, Logic and Scientific Method, London School of Economics, London, UK

**Keywords:** Ankle osteoarthritis, Contralateral feet, Control group, Pedobarography, Gait

## Abstract

**Background:**

Pedobarography offers dynamic information about the foot, but the interpretation of its large data is challenging. In a prior study it was shown that attention can be restricted to pedobarographic midfoot load data. We aim to verify this observation in ankle osteoarthritic and contralateral feet.

**Methods:**

We assessed both feet of 120 patients with end-stage ankle osteoarthritis (OA) and 35 healthy volunteers with AOFAS-score and dynamic pedobarography in barefoot condition. We introduce a new parameter, the Relative Midfoot Index (RMI), representing the depth of the midfoot weighted by the maximal force (MF) in the hindfoot and forefoot. Main outcome measures were the RMI, MF and contact times in the hindfoot, midfoot and forefoot. Ankle OA, contralateral and healthy feet were compared with ANOVA.

**Results:**

The RMI was significantly smaller in OA feet (0.65 ± 0.19) and contralateral feet (0.69 ± 0.15) than in healthy feet (0.84 ± 0.08, *p <* 0.0001). There was no significant difference between OA and contralateral feet. The RMI showed a correlation of 0.48 with the AOFAS score. Contralateral and OA feet were significantly different from healthy feet (*p <* 0.001) in all parameters except the hindfoot MF. An RMI <0.8 showed a positive predictive value of 80% and sensitivity of 78% for being unhealthy.

**Conclusion:**

The RMI assists the interpretation of pedobarographic parameters and provides a user-friendly indicator for unhealthy foot conditions with a cut-off value of 0.8. The contralateral feet of ankle OA patients differed significantly from healthy feet and are therefore not suitable as control group.

Level of Evidence: 3 case control study

## Background

Pedobarography is an established method to evaluate the function of feet and has been used to investigate many different kinds of pathologies as well as outcomes after surgeries [1–7]. Pedobarography offers dynamic information about the foot during the rollover process and therefore adds in important ways to static radiographic imaging. It is easily performed in the research setting, requires little time, and has low costs compared to a more complex three dimensional gait analysis.

These advantages contrast with the challenges posed by its data analysis. A standard pedobarography system such as the Novel Emed m/E system measures 18 basic and a number of optional parameters in 3 to 10 areas of interest as well as the total foot [8]. Such a measurement provides values of 72–198 (4 to 11 times 18) parameters for each foot [9]. These raw data are hard to interpret, and left unprocessed they provide no useful information. For this reason different authors have chosen to focus on selected parameters: average pressure [10], peak pressure [11], pressure time integral and contact time [1, 12]. However, it remains unclear whether selectively focussing on one of these is appropriate because no clear arguments have been given to choose one parameter over the other and there is worry the parameters have been picked to yield positive results. For this reason, the extraction of clinically useful information from pedobarographic data remains a challenge.

A solution to this problem has to meet a several conditions: First, a clear prescription has to be given about with which parameters to report. Second, the number of parameters has to be reduced: Often groups are compared with each other and with the 72–198 parameters from the Novel software, this would involve carrying out 72–198 t-tests for two groups, 216–594 for 3 groups (=3× 72 to 3×198), and 432 to 1188 for 4 groups (=6× 72 to 198). In practice this would be time consuming and would include the reporting of 5% of false positive test results as side effect (e.g. 22–59 false significant rest results in comparing 4 groups). Third, load parameters need to be normalized to body weight as any load parameter is directly dependent on the body weight, which distorts comparison between individuals.

To make pedobarograhy more user-friendly in daily clinical work, an easy-to-use parameter is needed. The first aim of this work was to develop and introduce such a parameter, which facilitates interpretation of a pedobarographic measurement. We call this parameter the Relative Midfoot Index (RMI). We show that this index is useful in the analysis of ankle osteoarthritis. To this end we examine patients with ankle osteoarthritis and compare them to healthy participants.

Another problem encountered with comparison and interpretation of pedobarographic data is that there exist two different ways for comparison of the affected feet: comparison with the unaffected contralateral foot [13–15] or the comparison with feet of healthy participants [16–18]. The first method assumes that contralateral feet can be regarded as healthy and that the foot problem on one side would not affect the other side. However, concerns about this assumption can be raised because clinical experience suggests that contralateral feet are affected by issues with the affected feet. For this reason healthy participants were added in this study, which allows for an evaluation of the assumption that contralateral feet can serve as healthy controls.

To summarise, the aims of this study are:

1. To introduce a new parameter called the Relative Midfoot Index (RMI) that facilitates interpretation of pedobarographic parameters and provides a user-friendly indicator for an unhealthy foot condition.
2. To study out the differences between ankle OA, contralateral and healthy feet by comparing their respective RMI, maximal force and contact time (in the hindfoot, midfoot and forefoot).
3. To define an RMI cut off value below which feet are considered unhealthy.
4. To compare contralateral non-affected feet with healthy feet to see whether contralateral feet are a suitable control group.
5. To investigate how the pedobarographic parameters correlate with the clinical outcome measured by the AOFAS-score.

## Methods

### Study participants

This study included 120 consecutive patients (54 female; 66 male; average age 59.5 years, SD ± 12.17) with symptomatic unilateral posttraumatic end-stage ankle osteoarthritis (OA) prior to surgery (total ankle replacement or arthrodesis) who were seen in the Department of Orthopaedics of the authors’ University hospital and 35 healthy volunteers (70 ft; 18 female; 17 male; average age 37.41 year (SD ± 12.36) from another hospital measured by the same team following the same protocol. The inclusion criteria for the OA group were a unilateral posttraumatic end-stage ankle OA with an indication for either ankle fusion or total ankle arthroplasty. Exclusion criteria were bilateral ankle injuries, primary ankle OA, inflammatory secondary ankle OA, rheumatoid ankle OA, Charcot ankle neuroarthropathy and patients showing an abnormal gait resulting from other reasons. Volunteers were recruited from the patients’ companions and included if they had no history of foot complaints or disorders, an unlimited walking capability (AOFAS score 100 points, American Orthopedic Foot and Ankle Society) [19] and no pathologies in clinical examination of the foot. The baseline demographics of the two groups are given in Table 1.

**Table 1:** Descriptive data of patients and healthy participants (OA: Osteoarthritis, SD: standard deviation)

	Ankle OA feet		Contralateral feet		Healthy feet	
	mean	SD	mean	SD	mean	SD
N	120		120		70	
Age (years)	59.52	12.17	59.52	12.17	37.41	12.36
Weight (kg)	80.40	15.21	80.40	15.21	72.66	14.45
MF-hindfoot (N)	469.91	149.52	520.22	123.95	516.10	105.29
MF-Midfoot (N)	208.13	120.38	195.81	100.99	86.41	51.60
MF Forefoot (N)	744.03	191.35	771.04	164.23	590.84	132.90
Contanct-Time Hindfoot (ms)	571.13	231.69	573.10	211.55	406.97	76.17
Contatct Time Midfoot (ms)	654.75	211.02	673.02	209.76	429.35	90.15
Contact Time Forefoot (ms)	890.54	242.76	808.45	241.51	584.85	72.66
RMI	0.654	0.192	0.696	0.146	0.844	0.083

All participants signed an approved informed consent form. The Ethical Review Board of the authors’ university gave approval to the study and the study was performed in accordance with the World Medical Association Declaration of Helsinki.

### Pedobarography

Gait analysis of all patients and healthy volunteers was performed using dynamic pedobarography (Novel emed m/E, St Paul, MN or EMED, Novel GmbH, Munich, Germany). The runway consisted of hard plastic and had a measuring plate with 2736 sensors (spatial resolution of 4 sensors/cm^2^). The dynamic foot load was measured with a frequency of 50 Hz. Participants performed a minimum of five walks per foot to make sure that the software had enough acceptable footprints.

**Fig. 1:**
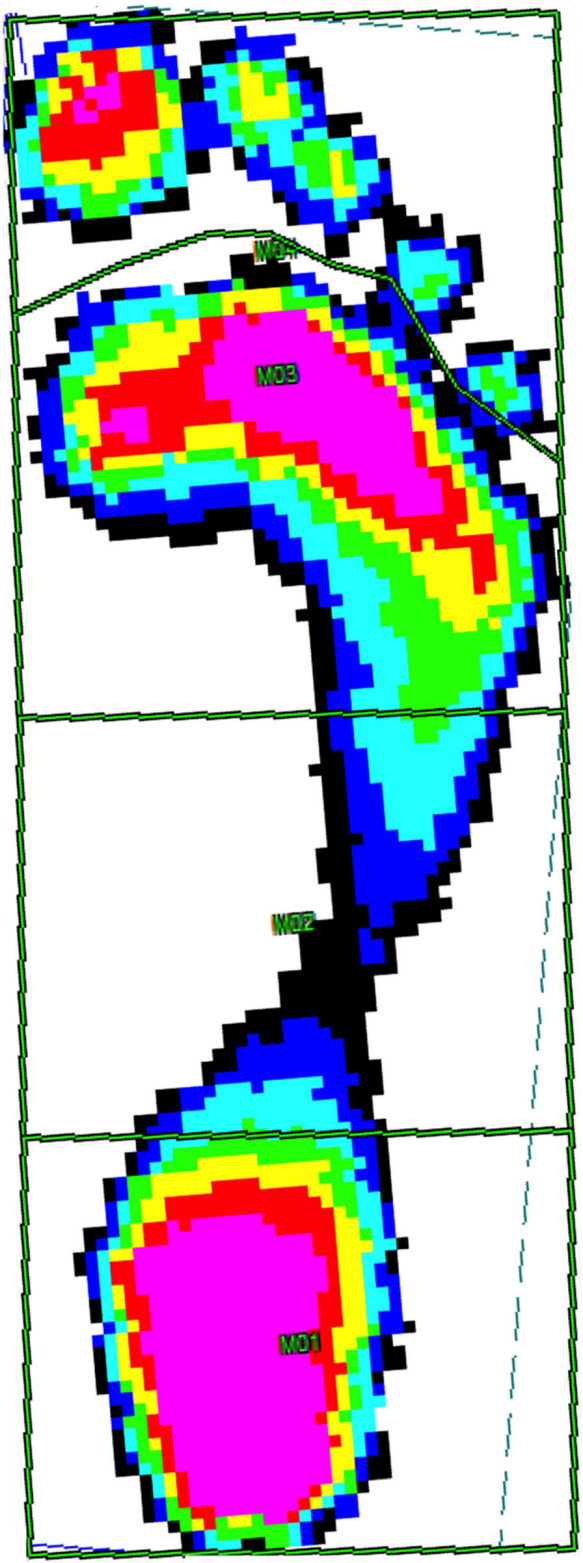
Four-area mask (hindfoot, midfoot, forefoot, toes) from the Novel scientific software (EMED, Novel GmbH, Munich, Germany). The heel-to-midfoot boundary was specified as 45% of length and the midfoot-to-forefoot boundary was defined as 73% of length [8]

Participants walked barefoot with normal steps and at their own chosen speed. They took five steps before and after walking onto/off the measuring plate in order to avoid effects of acceleration and deceleration [20]. Data were then analysed with the Novel scientific software using a four-area mask (hindfoot, midfoot, forefoot, toes, Fig. 1). The heel to midfoot boundary was specified as 45% of length and the midfoot to forefoot was defined as 73% of length [8]. The toe mask was excluded for two reasons: single toes may show high pressures and toes are not as important as the rest of the foot for the rollover process.

### Relative midfoot index

**Fig. 2:**
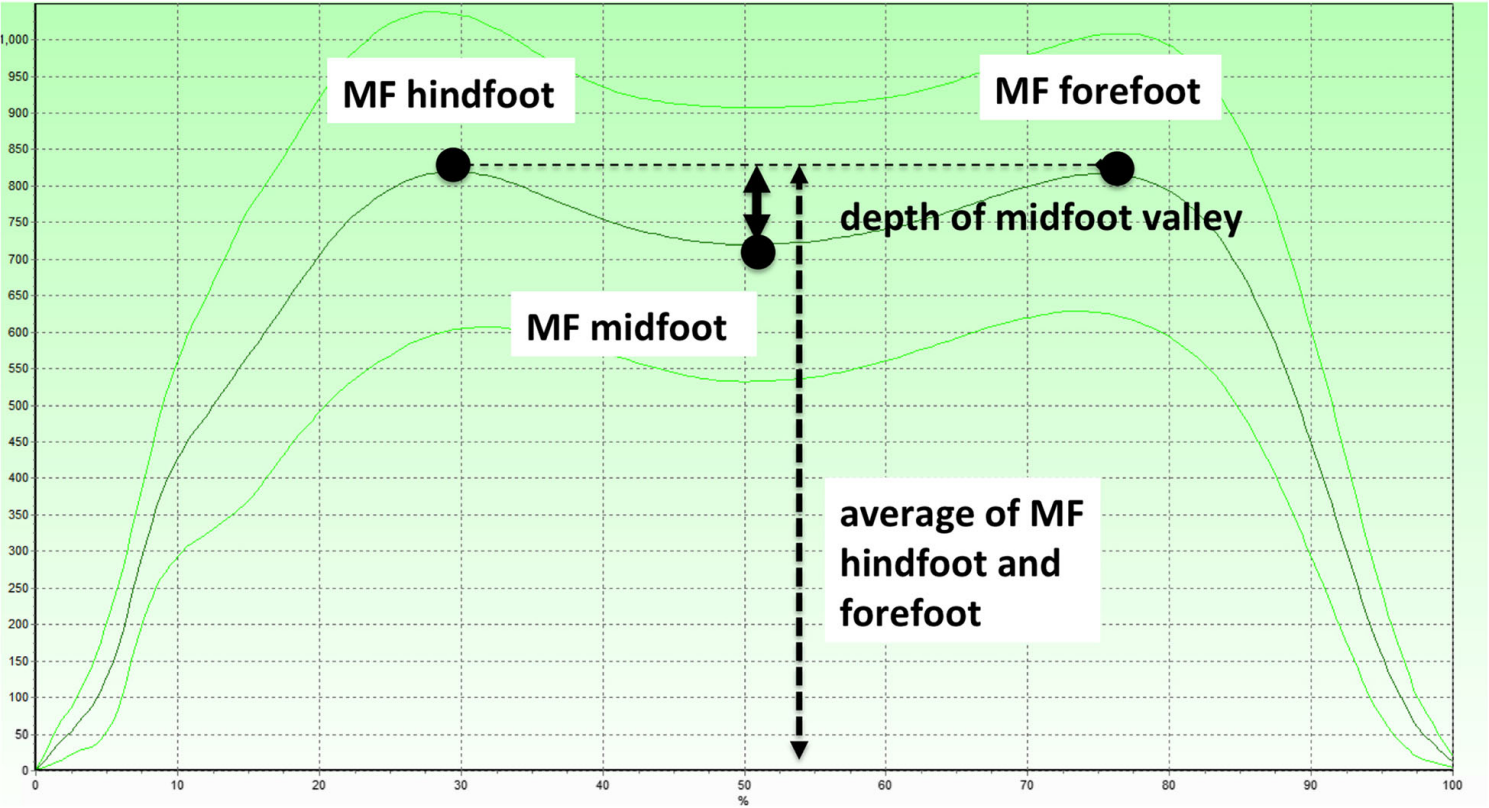
Relative midfoot index (RMI): The RMI is calculated by putting the depth of the midfoot valley in relation to the average of the MF in the hindfoot and forefoot

In an earlier study, the large number of pedobarographic parameters was reduced to 27 parameters, 9 each for hindfoot, midfoot, and forefoot, and aggregated into two clusters: One cluster of *rollover parameters*, describing the temporal motion of the foot over the ground from heel strike to toe off (containing the centre of pressure velocity, contact time, instants of maximal force (MF) and peak pressure (PP)) and one cluster of *load parameters* (MF and PP, integral of MF and PP) [9]. This reduction was crucial to make the data amenable to statistical analysis and to pave the ground for a clinical interpretation of results. The core result was that the cluster of load for the midfoot was the most important predictor to distinguish between healthy volunteers, ankle arthrodesis (AA) or tibiotalocalcaneal arthrodesis (TTC) and that the MF had the strongest correlation within this cluster [9]. Furthermore, MF is generally the parameter that provides most insight into gait mechanics because, unlike pressure, it is independent of local foot callosities or deformities. We therefore created a new parameter, which we call the Relative Midfoot Index (RMI) representing the depth of the midfoot valley of the force-time curve in relation to the amount of maximal force (MF) in the hindfoot and forefoot (Fig. 2):

$$ RMI=1-\frac{2M{F}_m}{M{F}_f+M{F}_h} $$

**Fig. 3:**
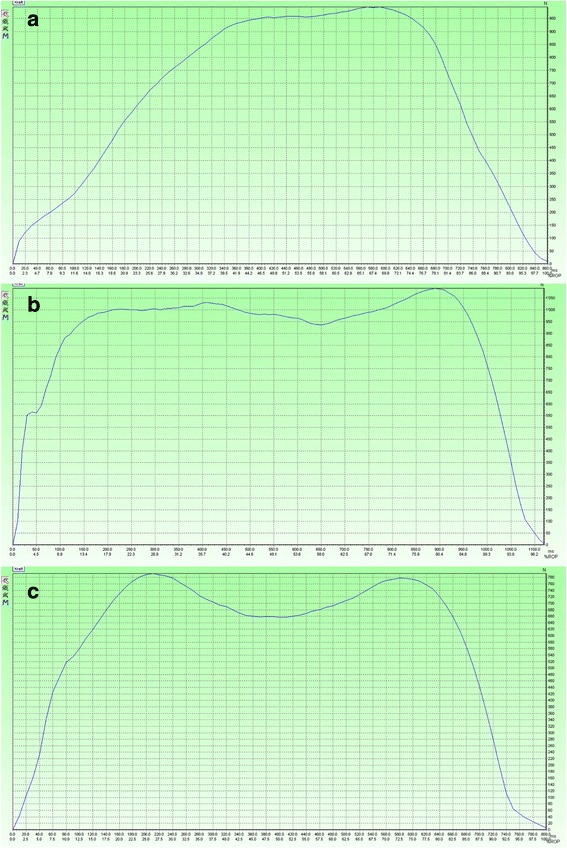
Maximal force curves of a foot with ankle osteoarthritis (**a**), of the contralateral unaffected foot of a patient with ankle osteoarthritis (**b**) and a healthy foot (**c**). These graphs show that the midfoot depression is small in the ankle-osteoarthritic foot (*biphasic pattern*) and deep in the healthy foot (*triphasic pattern*). Figure b shows that the unaffected foot has a pathologic gait pattern and is therefore not suitable for comparison

*MF*_*m*_, *MF*_*f*_, and *MF*_*h*_ are the MF for the midfoot, forefoot, and hindfoot respectively. In effect the RMI is the *MF*_*m*_ weighted by the average of *MF*_*f*_ and *MF*_*h*_: in normal triphasic gait the RMI is expected to be close to one (deep midfoot depression on force-time graphs) while in pathologic biphasic gait it is expected to be close to zero (flat midfoot depression on force-time graphs, Fig. 3) [16]. The RMI has the advantage that it is independent of body weight and walking speed, which both influence the absolute values. The RMI therefore allows for simple comparisons between individuals.

### Other outcome parameters

In order to standardize the report of pedobarographic data, we also report the MF (as representation of load) and the contact times (as representation of rollover) in the hindfoot, midfoot and forefoot. For the clinical assessment, the AOFAS hindfoot score [19] was recorded.

### Statistical analysis

Data from pedobarography was extracted into ASCII-files that were then transformed into Excel files (Microsoft Corp, Redmond, WA, USA) using a custom-made algorithm written in MATLAB ® (The MathworksInc, Natrick, MA, USA). STATISTICA ® Version 8.0 (StatSoftInc, Tulsa, OK, USA) was used for all analyses. For testing parametric data an ANOVA-analysis was made. *P* values less than 0.05 were considered significant and the level of significance P was corrected for multiple testing by dividing the level of significance by the number of tests (0.05/21 = 0.0024, Table 2). Pearson correlation co-efficients (*r*) were calculated; *r* values of less than 0.4 were considered poor, 0.4–0.6 as weak, 0.6–0.8 as moderate, and greater than 0.8 as good. To find out which value of the relative midfoot index is the cut off value to differentiate between healthy and unhealthy, the positive predictive value (PPV), negative predictive value (NPV), sensitivity and specificity were calculated.

**Table 2:** *P* values of the comparison of the three groups to each other: OA-Contralateral, OA-Healthy, Contralateral-Healthy (OA: Osteoarthritis, MF: Maximal Force). Significant values are marked in italic

	Ankle OA - Contralateral	Ankle OA -Healthy	Contralateral - Healthy
Age (years)	1.0000	0.0000	0.0000
Weight (kg)	1.0000	0.0020	0.0020
MF-Hindfoot (N)	0.0081	0.0495	0.9762
MF-Midfoot (N)	0.6109	0.0000	0.0000
MF-Forefoot (N)	0.4314	0.0000	0.0000
Contact-Time Hindfoot (ms)	0.9968	0.0000	0.0000
Contact-Time Midfoot (ms)	0.7371	0.0000	0.0000
Contact-Time Forefoot (ms)	0.7967	0.0000	0.0000
Relative Midfoot Index	0.0966	0.0000	0.0000

## Results

The RMI for the ankle OA group was 0.654 (SD ± 0.192), for the contralateral group 0.696 (SD ± 0.146) and for healthy participants 0.844 (SD ±0.083). The value for the RMI was significantly smaller in osteoarthritic feet compared to healthy feet (*p <* 0.0001). Contralateral feet also had a significantly lower RMI than healthy feet (*p <* 0.0001). There was no difference in the RMI of affected and contralateral feet. For all other parameters see Tables 1 and 2, Fig. 3.

As regards the cut-off, results showed that a relative midfoot index of 0.8 was the best compromise with a positive predictive value of 80% and sensitivity of 78% to determine whether a foot is healthy or not (Table 3). By way of comparison, for an RMI value of 0.7 the PPV was better (99%) but the sensitivity (60%) was too weak, and therefore many unhealthy feet would remain undetected (Table 3).

**Table 3:** Test statistics of the relative Midfoot Index (RMI) to define a cut-off value to differentiate between healthy and diseased (PPV: Positive Predictive Value, NPV: Negative Predictive Value)

RMI	<0.9	<0.8	<0.7	<0.6	<0.5
PPV	0.68	0.80	0.99	0.98	1
NPV	0.64	0.64	0.59	0.46	0.42
Sensitivity	0.91	0.78	0.60	0.33	0.2
Specifity	0.27	0.67	0.99	0.99	1

Looking at the MF and contact time in the hindfoot, midfoot and forefoot, we find that the values of all parameters except the hindfoot MF of both OA and contralateral feet differed significantly from the values of healthy feet (*p <* 0.001, Table 2).

The AOFAS score was 44.1 ± 16.84 points in ankle OA and 100 points in healthy participants as defined by the inclusion criteria. There was a weak correlation of 0.48 between the AOFAS score and the RMI. Furthermore the AOFAS score correlated weakly with the MF in the midfoot (*r =* 0.49), while it anticorrelated weakly with the contact time in the hindfoot (*r* = −0.42), the contact time in the midfoot (*r* = −0.52) and the contact time in the forefoot (*r = −*0.58, Table 4).

**Table 4:** Correlation of measured parameters with the AOFAS-Score of healthy participants and ankle OA patients (MF: Maximal Force)

Age (years)	−0.6029
Weight (kg)	−0.2972
MF-Hindfoot (N)	0.1172
Contact-Time Hindfoot (ms)	−0.4177
MF-Midfoot (N)	−0.4936
Contact-Time Midfoot (ms)	−0.5196
MF-Forefoot (N)	−0.3689
Contact-Time Forefoot (ms)	−0.5814
Relative Midfoot Index	0.4786

## Discussion

This study introduces a new parameter, the so-called relative midfoot index (RMI). The RMI facilitates the interpretation of a large number of pedobarographic paramaters. 120 patients with end-stage ankle OA and their non affected contralateral feet as well as 35 healthy volunteers (70 ft) were measured. The results showed that osteoarthritic feet and healthy participants had a significantly different RMI. A RMI <0.8 showed a positive predictive value of 80% and sensitivity of 78% to detect an unhealthy foot condition. Furthermore there were significant differences between contralateral feet and healthy feet. A correlation (*r =* 0.48) between AOFAS-score and RMI in healthy and ankle OA patients was found.

This study has several limitations: First, no matching for age and weight was made between the OA patients and the healthy participants, which resulted in the fact that the control group was lighter, healthier and younger. The same issue has been noticed also by other authors [21]. However as regards weight, the RMI ensures independency from weight for means of comparison, which successfully mitigates against this difficulty. As regards age, Bosch [11] observed a significant increase in the midfoot load in seniors compared to adults. One could therefore argue, that the measured differences are due to age and not osteoarthritis. Calculating the RMI with Bosch’s published data, we find an RMI for adults of 0.87, for 7-year olds 0.82 and for seniors of 0.79 (adults 31.9 ± 2.1 years, 7-year olds 7 ± 0.4 years, seniors 68.7 ± 3.2 years). The RMI of seniors is marginaly healthy, however aging is associated with degenerative changes and this was not an exclusion criteria in Bosch’s study, which influences the gait. Second, this study has been performed using pedobarography, which produces a large amount of data. This study has identified the relevant variable and so future studies can use simpler methods such as a force plate to gather data in a targeted manner.

The RMI is a useful parameter as it is independent of weight and walking speed, which both affect the absolute force values. Furthermore it was built on the observation of a prior study [9], which recognized the cluster of load of the midfoot as the best parameter to distinguish between a healthy and a fused ankle. In this connection it is interesting to note that other authors also focussed on the midfoot load: Piriou compared healthy and osteoarthritic feet and recognised that the ground reaction force was flattened in the OA feet [21]. Mitternacht noticed a flattened ground reaction force after a calcaneus fracture and a resulting change in the rollover process [16]. These findings are in line with our own. To the best of our knowledge there are no further publications describing the flattened force-time graph in diseased feet. Further studies are needed to define which foot and ankle pathology leads to which pedobarographic alterations in the affected foot.

The other aim of the study was to figure out if there was a difference between contralateral feet and healthy feet. In the literature both feet were used as a “healthy” standard against which diseased feet were judged. Some authors worked with a healthy control group [16–18, 22], while others regarded the contralateral feet as the control group [12–15]. The latter assumes that collateral feet behave like healthy feet. The current study found that there is a significant difference between contralateral feet and truly healthy feet for all parameters analysed in this study except the hindfoot maximal force. Therefore the choice of contralateral feet as healthy controls is unwarranted.

This study aimed to find an association between the RMI and the clinical outcome measured by the AOFAS-score. We found a positive correlation between RMI and AOFAS-score (*r =* 0.48). In the past, other studies have also tried to identify correlations between AOFAS score and pedobarographic parameters. In a study comparing ankle- and tibiotalocalcaneal arthrodesis to healthy participants we also found a correlation between different midfoot load parameters (MF, PP, integrals of MF and PP) summarized as “midfoot index of load” and the AOFAS-score [9]. Schuh found a significant correlation between the AOFAS score and the loading parameters of the medial midfoot after surgery of posterior tibial tendon dysfunction [23]. However the reported correlation coefficients were between −0.29 and −0.36, which are values that we consider not even weak. Rammelt realised, that a higher AOFAS-score is associated with a higher pressure time integral in the whole foot, but without reporting a correlation coefficient [12]. Burns noticed a correlation between the pressure time integral and foot pain (*r =* 0.49) in cavovarus feet [1], which is comparable to our result. In rheumatoid feet, Schmiegel detected an increase of the average pressure together with the severity of the impairment in the Health Assessment Questionnaire in three groups, but no correlation coefficient was calculated [10]. In summary, there were only very weak correlations between pedobarographic parameters and clinical findings.

## Conclusion

A RMI < 0.8 is an easy-to-use indicator of an unhealthy gait. This makes the RMI a helpful clinical tool to reach a quick first assessment of the condition of a foot. Since the relative midfoot index is independent of body weight and walking speed it can also be used for interindividual comparison. However, since the index has been tested on ankle osteoarthritis only, it remains an open question whether it also provides a useful indicator for other pathologies. We also showed that the contralateral foot doesn’t act like a healthy foot. As a consequence, contralateral feet are not suitable for comparison and cannot be used as healthy controls.

## Clinical relevance

This paper emphasises that pedobarographic results should be reported in a standardised format. The MF serves as a representation of everything having to do with load, and the contact time represents everything in connection with the rollover process. Second, to facilitate interpretation of pedobarographic parameters in the clinical setting, the RMI has been introduced. The advantage of the RMI is its independence of walking speed and bodyweight, which makes it suitable for inter-individual comparison. The RMI helps to distinguish between healthy triphasic gait and unhealthy biphasic gait with a cut off value of 0.8. We also showed that the contralateral foot doesn’t act like a healthy foot. As a consequence, contralateral feet are not suitable for comparison and cannot be used as healthy controls.

## Abreviations

AA: Ankle arthrodesis
AOFAS: American Orthopedic Foot and Ankle Society
MF: Maximal Force
NPV: Negative Predictive Value
OA: Osteoarthritis
PP: Peak pressure
PPV: Positive Predictive Value
RMI: Relative Midfoot Index
SD: Standard deviation
TTC: Tibio-Talo-Calcaneal arthrodesis
